# Evaluation of the impact of heat on wheat dormancy, late maturity α-amylase and grain size under controlled conditions in diverse germplasm

**DOI:** 10.1038/s41598-020-73707-8

**Published:** 2020-10-20

**Authors:** Jose M. Barrero, Luciana Porfirio, Trijntje Hughes, Jing Chen, Shannon Dillon, Frank Gubler, Jean-Philippe F. Ral

**Affiliations:** 1 CSIRO Agriculture and Food, Black Mountain Science and Innovation Park, Canberra, ACT Australia; 2 CSIRO Oceans and Atmosphere, The Climate Science Centre, Black Mountain Science and Innovation Park, Canberra, ACT Australia; 3 Chengdu Institute of Biology, Chinese Academy of Science, Chengdu, Sichuan China

**Keywords:** Plant breeding, Plant genetics, Plant physiology, Plant sciences, Climate sciences

## Abstract

In the Australian wheat belts, short episodes of high temperatures or hot spells during grain filling are becoming increasingly common and have an enormous impact on yield and quality, bringing multi-billion losses annually. This problem will become recurrent under the climate change scenario that forecast increasing extreme temperatures, but so far, no systematic analysis of the resistance to hot spells has yet been performed in a diverse genetic background. We developed a protocol to study the effects of heat on three important traits: grain size, grain dormancy and the presence of Late Maturity α-Amylase (LMA), and we validated it by analysing the phenotypes of 28 genetically diverse wheat landraces and exploring the potential variability existing in the responses to hot spells. Using controlled growth environments, the different genotypes were grown in our standard conditions until 20 days after anthesis, and then moved for 10 days into a heat chamber. Our study showed that our elevated temperature treatment during mid-late filling triggered multiple detrimental effects on yield and quality. We observed a reduction in grain size, a reduction in grain dormancy and increased LMA expression in most of the tested genotypes, but potential resistant lines were identified for each analyzed trait opening new perspectives for future genetic studies and breeding for heat-insensitive commercial lines.

## Introduction

Climate change has already influenced the patterns of agricultural production^1–4^ and about a third of the annual variability in agricultural yields is caused by climate variability^5,6^. The interaction between climate variability and climate change threatens the sustainability of traditional agricultural systems^7,8^. In the case of wheat, the consequences of heat stress include premature leaf senescence, reduced photosynthesis, reduced seed set, reduced duration of grain-fill, reduced grain size, and reduced grain yield^9^. The effects of heat impacting wheat yield and quality are well known and have been previously reported^10^. Those effects can be seen even after short period of heat or hot spells^11^, which are enough to influence the grain filling stages leading to lower yield and quality^12–14^. Interactions between heat and water availability or nitrogen supply have also been studied in the context of grain filling showing complex responses in both controlled environments and field experiments^15–18^. Current climate change predictions are forecasting an increase in temperature and of extreme weather events^19^ that will increase the number of hot spells per season^20^.

Recent field studies have found an enormous yield reduction for each day with maximum temperature over 30 °C both during and after anthesis^11^. Although many studies focused of grain size and yield^17,21^, high temperature may also have an impact of grain quality factors thus affecting severely the dough properties^13,14,16^. Fewer heat studies are available for two other traits affecting the quality and marketability of wheat and known as Pre-Harvest Sprouting (PHS) and Late Maturity α-Amylase (LMA). Although PHS and LMA are genetically and mechanistically independent, for them to occur specific environmental triggers are needed, and temperature has a major role in both cases^22,23^.

Several studies have evaluated the performance of different wheat cultivars in the presence of heat and some genetic variability has been reported^10,11,24,25^. Because of that variability the selection and breeding for heat tolerance is possible and even the genetic engineering of heat tolerance has been discussed^10,21^. However, the timing of heat stress during different stages of grain development will impact on particular molecular mechanisms and processes, so it is important to accurately define the window of heat stress targeted for genetic improvement. While several previous studies have mainly focussed in pre-anthesis or early filling stages, less work has been done during mid-late grain filling. Short periods of high temperatures during late maturation are already a recurrent problem in the Australian wheat belts, but so far, no comprehensive genetic analysis of the resistance to hot spells has been performed. As a first step to fill this knowledge gap, we developed a robust heat stress method using controlled environment and we used a core collection of 28 diverse wheat landraces representing global genetic diversity to explore the genetic variability existing in the responses to hot spells. Three important traits were examined: grain size, grain dormancy and the presence of LMA. Our study showed that ten days of heat stress during mid-late filling can reduce yield by reducing grain size by 20% in average. In addition, we found a striking reduction or loss of grain dormancy, thus increasing the risk of PHS. Finally, we found an increase in LMA, which will downgrade grain quality thus reducing its marketability. Importantly, we were able to identify lines that were resistant to heat for each of the examined traits. This study opens the door for mapping the genes responsible for the heat resistance during grain filling and for the breeding of heat-insensitive commercial lines.

## Results

### Analysis of historic temperature data and future prediction

**Figure 1:**
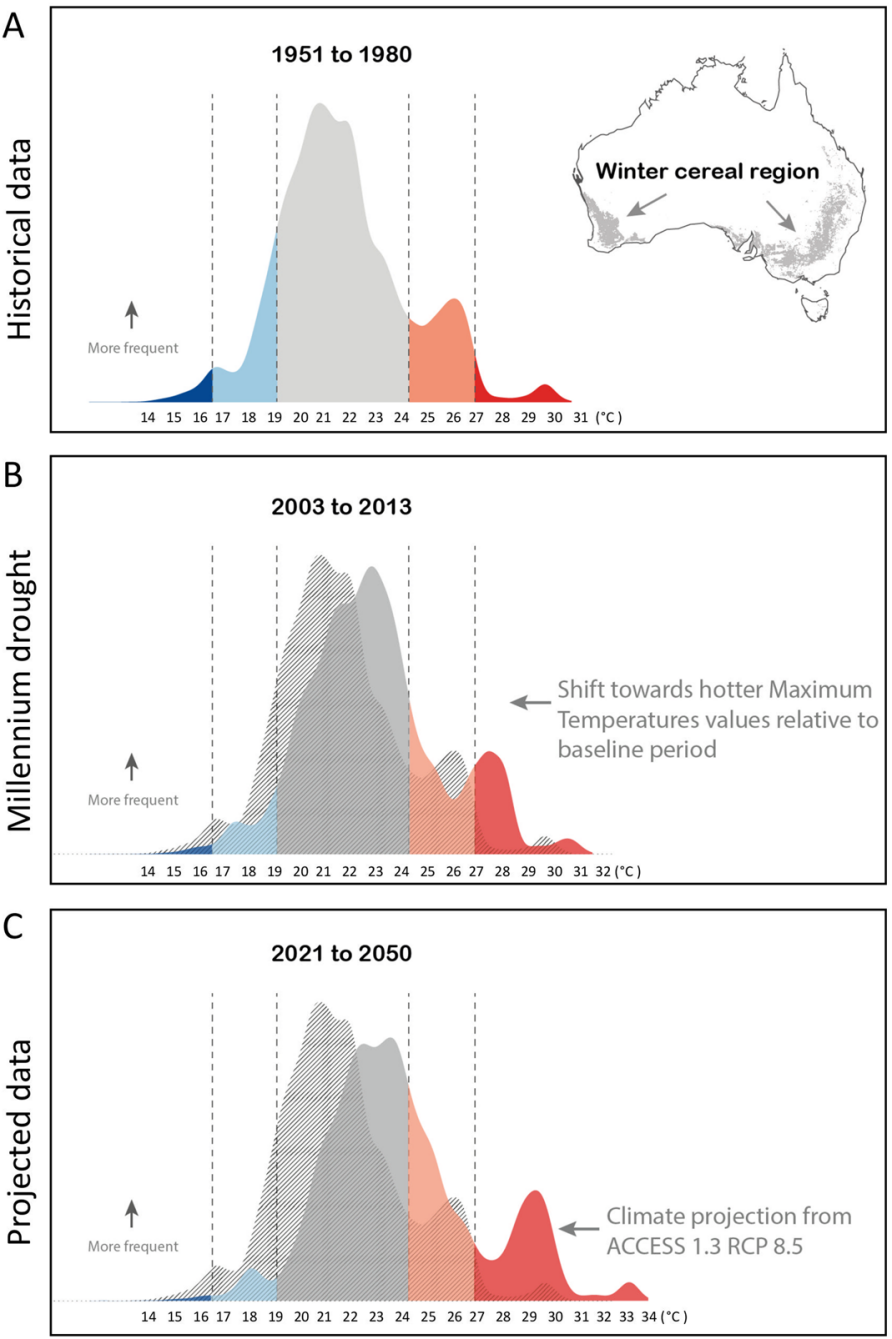
Shift in maximum temperatures during grain filling in the Australian wheat belt. Data of maximum temperatures for the period of September–October for the winter cereal region in Australia (grey area on the map). The colours and hatched vertical lines indicate values that depart one or two standard deviations from the baseline period mean (21.7 °C). **(A)** The curve-bells show climatologies of maximum temperature for the baseline period (1951–1980). The light red area represents high temperatures between 1 and 2 standard deviations and the red area represents temperatures over 2 standard deviations. The light blue area represents low temperatures between 1 and 2 standard deviations and the blue area represents temperatures below 2 standard deviations. The map was obtained from the Australian Land Use and Management (ALUM) Classification system version 8 (https://data.gov.au/dataset/ds-dga-a7d50fb8-b353-4bb4-a7ca-1a2f38f44abc/details), selecting only the pixels classified as ‘winter cereals’ using the Raster R Package. **(B)** Climatologies of maximum temperatures for the period 2003–2013, which includes the period known as Millennium drought. **(C)** Future projections of maximum temperatures for the period 2021–2050 from the Australian Earth System Model, Access 1.3, based on the high carbon emissions Representative Concentration Pathway scenario (RCP 8.5).

To understand the risk that high temperatures pose to wheat during mid-late grain filling in Australia, we performed an analysis of the maximum temperatures observed specifically in the Australian wheat belt during the months of September–October (Fig. 1). Firstly, we analyzed historical weather data from 1951 to 1980 and used it as a baseline for comparison for later periods (Fig. 1A). During 1951–1980 the number of extremely hot days (above 27 °C, two standard deviations over the mean) represented a small fraction of the total. When we examined a contemprary temperature dataset from 2003 to 2013 including the period referred in Australia as the “Millennium drought”, (an unusual period of drought and high temperatures), we observed that the percentage of very hot days increased significantly (Fig. 1B). Finally, we used a climate projection from ACCESS model^26^ 1.3 (RCP 8.5) to evaluate the future incidence of heat from 2021 to 2050 (Fig. 1C) and we found that the percentage of very hot days will continue to increase further, and that the occurrence of Millennium drought-like conditions would increase. Our analysis indicates that maximum temperatures in the Australian cereal region are getting higher, and that the hot spell occurrence is predicted to become more frequent.

### Effect of heat shock on grain dormancy

A core collection of 28 wheat landraces selected from diverse geographical origins and agricultural conditions (Table 1) was used to analyze the impact of hot spells during grain filling. Three commercial wheat cultivars with different grain characteristics and dormancy habits were included for comparison: AcBarrie (Canadian red wheat with the 4AL dormancy QTL^27^), Yitpi (Australian white wheat with the 4AL QTL^28^) and Mace (Australian white wheat). Young seedlings were planted into pots in growth cabinets and grown under our standard control conditions (20 °C at midday) as described in the Methods section. In one of the cabinets the plants stayed there until maturity. Differences in flowering time in this population were up to 20 days. As plants reached physiological maturity, ripened spikes from two main tillers were harvested from each genotype and stored in the freezer until the whole population was harvested. In a second adjacent growth cabinet, the same population was grown with the same experimental design and with the same environmental conditions. In this cabinet, up to three spikes from every plant were tagged at anthesis. At 20 days after anthesis the plants were moved for 10 days into a third cabinet where they were exposed to the heat shock treatment (36 °C at midday). After that the plants returned to the original cabinet and the tagged spikes were harvested at maturity and stored in the freezer until all genotypes were exposed to heat in the same way. The conditions of the control and heat treatments were chosen based on previous work on the effect of heat on grain dormancy, where it was demonstrated how this heat treatment can supress the major dormancy QTL in wheat^28^.

**Table 1:** Description of the wheat landraces and the commercial cultivars used in this work.

AUS	Acc_id	Name	Country	Description
4207	104204	BEYROUTH 4	Lebanon	Facultative
5163	105160	PALESTINE 1	Jordan	Spring
5266	105263	PERSIA 102	Iran	Hard/spring
5529	105526	PORTUGAL 174	Portugal	Facultative
6989	106986	CItr 4302	Iran	Tan/spring
7116	107113	BIHAR 119	India	Tan/spring
9532	109529	AUS 9532	Ethiopia	Spring
13129	113126	ZOCO DE YEBEL HEBIL	Morocco	Spring
13191	113188	BISKRI	Tunisia	Facultative
14973	114970	NW7A	Nepal	Red/Fac/winter
17953	117950	HAYNALDIA VILLOSA: ACM 1538	Former Soviet Union	Fac/winter
19122	119119	AMC 70	Iraq	Spring
26431	131109	CRETE 15	Greece	Spring
27289	131493	AFGHANISTAN 82	Afghanistan	Fac/winter
27464	131666	CROATIA 10	Yugoslavia	Fac/winter
27512	131714	GEORGIA W57143	Georgia	Spring
27524	131723	HUNGARY 4	Hungary	Spring
27857	131783	INDIA 316	India	Spring
28008	131934	SINAI 1	Egypt	Spring
28232	132158	SMYRNA 8	Turkey	Spring
28248	132174	VARNA 7	Bulgaria	Fac/winter
38401	172857	IG 40866	Syria	Spring
38475	172931	IG 93970	Algeria	Spring
38554	173010	IG 126264	Armenia	Spring
38592	173048	IG 138692	Bosnia-Herzegovina	Fac/winter
38632	173088	IG 141139	Kazakhstan	Spring
38703	173159	IG 141227	Tajikistan	Spring
38903	173790	IG 41440	Pakistan	Fac/winter
165	AGG36672WHEA	MACE	Australia	White/spring
165	AGG39193WHEA	ACBARRIE	Canada	Red/spring
165	AGG30492WHEA	YITPI	Australia	White/spring

**Figure 2:**
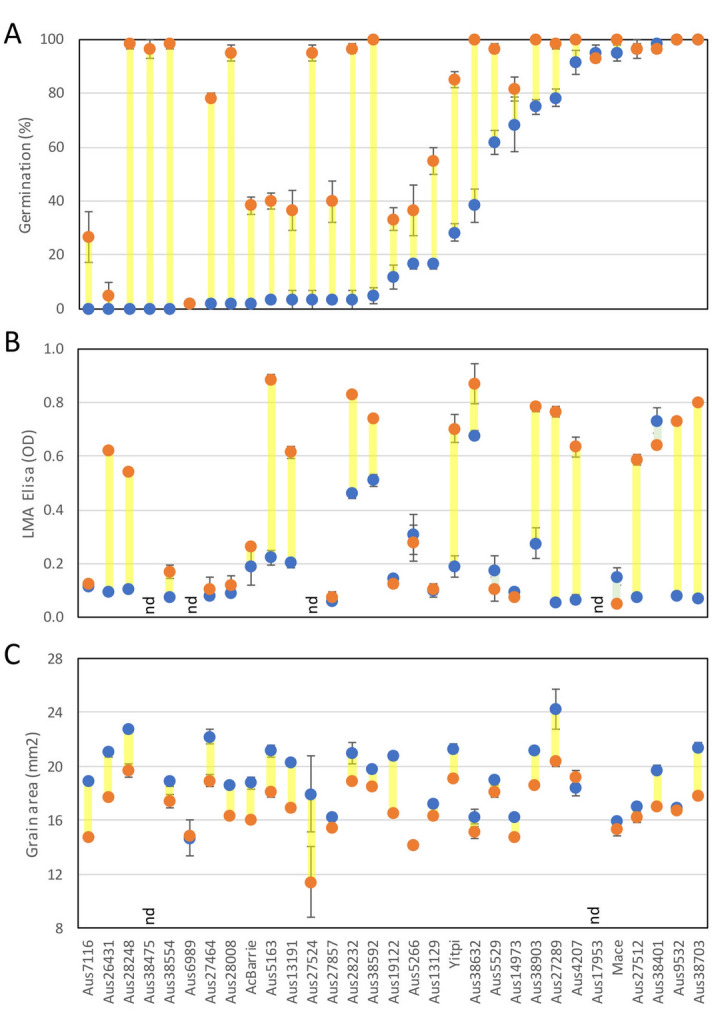
Impact of hot spells on grain dormancy, LMA and grain size. **(A)** Germination percentage of fresh-harvested samples. **(B)** LMA expression based on enzymatic assays. **(C)** Grain size analysis. Blue circles represent the values of the control samples. Orange circles represent heat stressed samples. Yellow lines were added to facilitate the comparison between treatments. Mean values with their SEs are shown. nd: not determined.

Germination assays were conducted to analyse the impact of the heat stress on grain dormancy of control and heat-treated samples (Table 2). From the commercial varieties we tested, AcBarrie was the most dormant but lost about 40% of the dormancy after the heat treatment. Yitpi had intermediate dormancy and became non-dormant after heat treatment. Mace did not show any dormancy under the control conditions (Fig. 2A). The landraces grown under the control treatment showed a large range of germination (from 0 to 100%), indicating a large variability in grain dormancy in this collection (Fig. 2A). In the heat-treated samples, the observed germination increased in most lines (Table 2). Some genotypes that were strongly dormant when grown under the control treatment lost all the dormancy in response to the heat stress (i.e. Aus28248), and some genotypes were able to retain some dormancy. Very interestingly we were able to identify two genotypes that were insensitive to the heat treatment and maintained their dormancy levels (i.e. Aus26431 and Aus6989).

**Table 2:** Classification of the landraces based on Germination %, LMA expression and grain size reduction.

Line name	Germination (%)		LMA expression		Grain size (mm^2^)		
	Control	Heat	Control	Heat	Control	Heat	Size reduction %
**Aus7116**	**0.0**	*26.7*	**0.1**	**0.1**	18.9	14.8	***21.9***
**Aus26431**	**0.0**	**5.0**	**0.1**	***0.6***	21.0	17.7	***15.8***
**Aus28248**	**0.0**	***98.3***	**0.1**	***0.5***	22.8	19.7	***13.5***
**Aus38475**	**0.0**	***96.7***	Nd	nd	nd	nd	nd
**Aus38554**	**0.0**	***98.3***	**0.1**	**0.2**	18.9	17.4	*7.6*
**Aus6989**	**1.7**	**1.7**	Nd	nd	14.7	14.8	**-0.8**
**Aus27464**	**1.7**	***78.3***	**0.1**	**0.1**	22.2	18.9	***14.7***
**Aus28008**	**1.7**	***95.0***	**0.1**	**0.1**	18.6	16.3	***12.4***
**AcBarrie**	**1.7**	*38.3*	**0.2**	*0.3*	18.8	16.0	***14.5***
**Aus5163**	**3.3**	*40.0*	*0.2*	***0.9***	21.1	18.1	***14.6***
**Aus13191**	**3.3**	*36.7*	*0.2*	***0.6***	20.3	16.9	***16.6***
**Aus27524**	**3.3**	***95.0***	nd	nd	17.9	11.4	***36.4***
**Aus27857**	**3.3**	*40.0*	**0.1**	**0.1**	16.3	15.4	*5.3*
**Aus28232**	**3.3**	***96.7***	***0.5***	***0.8***	21.0	18.9	***10.0***
**Aus38592**	**5.0**	***100.0***	***0.5***	***0.7***	19.8	18.5	*6.5*
**Aus19122**	*11.7*	*33.3*	**0.1**	**0.1**	20.8	16.5	***20.6***
**Aus5266**	*16.7*	*36.7*	*0.3*	*0.3*	14.1	14.2	**-0.3**
**Aus13129**	*16.7*	***55.0***	**0.1**	**0.1**	17.2	16.4	**5.0**
**Yitpi**	*28.3*	***85.0***	**0.2**	***0.7***	21.3	19.1	***10.2***
**Aus38632**	*38.3*	***100.0***	***0.7***	***0.9***	16.3	15.1	*7.0*
**Aus5529**	***61.7***	***96.7***	**0.2**	**0.1**	19.0	18.1	**4.3**
**Aus14973**	***68.3***	***81.7***	**0.1**	*0.1*	16.2	14.8	*8.9*
**Aus38903**	***75.0***	***100.0***	*0.3*	***0.8***	21.2	18.6	***12.1***
**Aus27289**	***78.3***	***98.3***	**0.1**	***0.8***	24.2	20.4	***16.0***
**Aus4207**	***91.7***	***100.0***	**0.1**	***0.6***	18.4	19.2	**-4.4**
**Aus17953**	***95.0***	***93.3***	Nd	nd	Nd	nd	Nd
**Mace**	***95.0***	***100.0***	**0.2**	**0.1**	16.0	15.3	**4.2**
**Aus27512**	***96.7***	***96.7***	**0.1**	***0.6***	17.0	16.2	**4.7**
**Aus38401**	***98.3***	***96.7***	***0.7***	***0.6***	19.7	17.0	***13.7***
**Aus9532**	***100.0***	***100.0***	**0.1**	***0.7***	16.9	16.7	**1.1**
**Aus38703**	***100.0***	***100.0***	**0.1**	***0.8***	21.4	17.8	***16.7***

The different genotypes were classified into three categories for each trait: bold (< 5% germination, < 0.2 LMA, or < 5% seize reduction), italic (> 5% to < 50% germination, > 0.2 to < 0.5 LMA, or > 5% to < 10% size reduction) and bold italic (> 50% germination, > 0.5 LMA, or > 10% size reduction).

### Effect of the heat stress on the expression of LMA

The grains from 24 landraces and commercial varieties were analyzed for their LMA expression (Fig. 2B, Table 2). Amongst commercial varieties, only Yitpi showed an accumulation of high-pI α-Amylase in response to heat stress with a post-treatment OD above 0.5, the cut-off for LMA susceptibility^29^. Among the tested landraces, Aus28232, Aus38592, Aus38632 and Aus38401 showed presence of LMA (OD above 0.5) in the control treatment suggesting a constitutive expression of LMA. The other 20 landraces displayed very low expression of LMA under control treatment (OD from 0.1 to 0.3) but after the heat shock treatment the LMA expression was significantly increased in 12 lines (OD from 0.6 to 0.9). We were able to identify 8 landraces in which the heat shock did not significantly induce expression of LMA (i.e. Aus7116).

### Effect of heat shock on grain size

Grain dimensions were calculated from samples collected from the control and from the heat-treated plants. Grain area, length and width were measured (Fig. 2C and Supplementary Fig. 1). An average decrease of 20% in grain area was observed after the heat shock treatment in all lines (Table 2). From the commercial varieties, AcBarrie and Yitpi both suffered a decrease in seed size while Mace did not. Regarding the landraces we found four which maintained their grain sizes after the heat shock (i.e. Aus6989). An interesting observation is that the four landraces that were insensitive to heat were the genotypes with the smallest grain area. The reduction in grain area that we observed in most of the genotypes was due to a reduction in the grain width and not in grain length. Grain length was only significantly reduced in Aus27524 (Supplementary Fig. 1).

### Genetic diversity evaluation of the landraces

**Figure 3:**
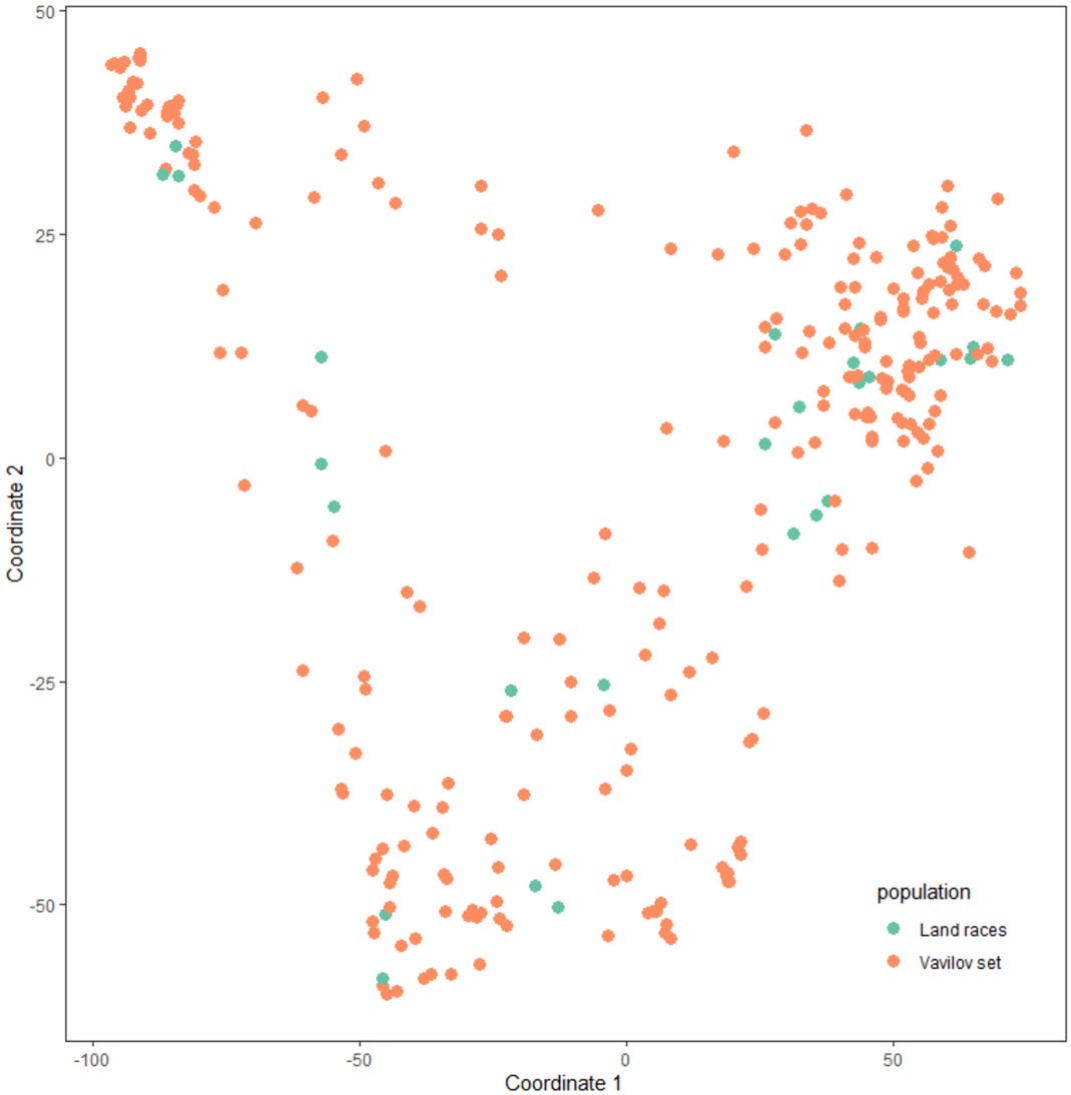
Genetic diversity analysis of the landraces in comparison to the Vavilov collection. Representation of the PCA of the genotyping results for the core landrace collection and the Vavilov diversity panel. The first two orthogonal axes (coordinates) produced following PCA on pairwise distance among varieties are plotted, illustrating the spread of diversity captured in the broader global collection (Vavilov set) relative to the landraces chosen for this study. The diversity seen in the landraces covers proportionally the diversity present in the Vavilov panel.

To evaluate the genetic diversity present in our core collection of landraces, we compared them with a well-established wheat diversity panel representing broad genetic variability, the Vavilov collection^30^. Both sets, our landraces and the Vavilov lines, were genotyped with the Illumina iSelect 90K SNP array^31^ and compared by clustering analysis (Fig. 3). The spread of point coordinates for each variety following visualisation of multivariate axes produced by the MDS analysis indicate that the 28 landraces selected for this study represent a sample of the diversity spanning the larger and denser Vavilov collection, and supports the use of our core collection for initial exploration of genetic variation in response to heat stress in wheat.

## Discussion

Wheat is the most widely cultivated crop worldwide, contributing one-fifth of the total calories consumed by humans. Australia is the 4th world wheat producer and the 5th biggest exporter, so the impact that increasing temperatures in this country will have in this crop is likely to impact global wheat trade^4^. Current climate modelling predicts an increase in the number of hot days during the grain filling period, so identification of germplasm resistant to hot spells is critical for wheat breeders not only in Australia but worldwide^32,33^.

Previous studies have found that a 3–5% reduction in yield (190 kg/ha) can occur for every degree increase in average temperature above 15 °C^34–36^. More recent field studies have found a reduction of 302 kg/ha per degree for each day with a maximum temperature in excess of 30 °C during anthesis, and a reduction of 161 kg/ha per degree for each day with a maximum temperature in excess of 30 °C during grain fill^11^. Prior to anthesis the heat stress causes reduced fertility thus reducing grain number, whilst heat stress during grain filling causes reduced grain size. Although both effects are very important, the impact of high temperatures during grain filling is more frequent due to the rising temperatures towards the end of the cropping season which have a severe effect on yield and quality^12^. The effect of heat stress on the components of grain weight has been extensively analysed in wheat and some genetic variability was found^37^. The authors found that heat stress reduced both the duration and the rate of grain growth. Starch and protein synthesis are also reduced under heat stress^16^, but a few thermotolerant genotypes have been identified. Regarding hot spells, it has been reported that just 3 days of heat stress during filling can reduce grain weight from 11 to 34%^12^. Our heat stress treatment was more severe to guarantee a strong impact on the diverse panel. This treatment showed that 10 days of heat was able to impact on grain size by reducing it by 20% on average in our core landraces. This results are in accordance with a recent study that used even a stronger treatment of 14 days with temperatures over 40 °C in the field and reporting a 57.3% grain yield reduction compared to a close‐by non‐stressed control^38^. As expected, our mid-late heat treatment impacted primarily on grain filling as indicated the decrease in grain width, but not length (except for Aus27524). We were able to identify some variability in the responses to heat in our diverse population for grain size stability. Genotypes with smaller grains under the control treatment were less impacted by the heat treatment, indicating a potential link between grain size and grain size stability. However, Aus4207, which has an average grain size, showed no size reduction in response to heat, indicating that heat stability can be retained in genotypes with adequate grain size.

Regarding grain quality issues, both PHS and LMA affect the quality of products made from the flour, thereby lowering the value of crops to end users such as bakers, causing significant economic losses to the grain industry^23,39^. Both PHS and LMA could be linked to a decrease in dough strength that has been associated with heat stress in previous studies^13,14^. It has been estimated that only 5 to 10% contamination by LMA or PHS-affected grains is enough to lower the value of bulk commodities below acceptable levels. PHS is the germination of the grain in the head before harvest. It is caused by a combination of low dormancy and environmental conditions such as high moisture. PHS is promoted under conditions of prolonged dampness or rain prior to harvest. Premature germination triggers the production of α-Amylases and proteases which break down the starch and gluten reserves in the endosperm. Flour from sprouted grain will fail the Hagberg Falling Number test^40^ which is the standard method to grade grain quality. It is estimated that PHS can produce losses exceeding US $1 billion per annum worldwide^41^.

Although moisture and cool temperature at harvest ripe is the critical factor triggering PHS, the temperature during grain filling have a major influence on determining grain susceptibility to PHS^42,43^. Cool temperatures during late maturation are associated with the development of deeper grain dormancy which then provide stronger protection against PHS^44^. On the other side, high temperatures during maturation results in grain with weaker dormancy making them prone to PHS^44^. Grain dormancy is the main genetically controlled factor that directly influences PHS^45^. In barley similar responses are observed and temperature-sensitivity windows for PHS susceptibility have been described during grain maturation^46^. Those windows of sensitivity can vary from cultivar to cultivar, but they always appear during mid-late grain filling. In our experiment we have targeted a small window during mid-late grain filling, which had a profound effect on grain dormancy. Under our control condition about half of the landraces that we tested displayed high dormancy with less than 5% germination, but after the heat treatment only two of them could be classified as dormant, with the other losing from 20 to 100% of their dormancy levels (Table 2). The sensitivity to heat for this trait is remarkably variable and we could identify lines with temperature-resistant dormancy (TRD) and lines with temperature-sensitive dormancy (TSD). Previously we reported that even the presence of the major 4AL QTL for grain dormancy does not provide TRD and that only 5 days of heat are able to supress grain dormancy in wheat genotypes carrying the 4AL QTL^28^. In agreement to that, two of the commercial cultivars that we have tested, Yitpi and AcBarrie, carry that QTL and displayed TSD. In Yitpi the germination increased after heat from 20 to 80% germination and in AcBarrie from 0 to 40%. The molecular mechanism/s providing TRD are not known but it appears that several factors could contribute. For example, the grain colour can have a role in providing some protection^47^. It will be also interesting to analyse the alleles of major known dormancy genes in the different landraces. Previous reports have shown that dormancy development under different temperatures is associated with the expression of the *TaPM19-A1* gene^28^ and the *MOTHER OF FT AND TFL1* gene (*MFT*)^48^. The sequence and expression of other important genes such us the *mitogen-activated protein kinase kinase 3* (*MKK3*) gene^49^ or *VIVIPAROUS1 (VP1*)^50^ could be analysed as well. It is also possible that new genes not previously reported are critical for the heat resilience and the development of TRD.

LMA was reported for the first time in Australia in 1993^51^. LMA is a random accumulation of a single type of α-Amylase (high-pI amylase) in the aleurone layer of developing grain^52^. This α-Amylase remains localised in the aleurone layer until grain maturity but can lower the marketability of grain after milling because it impacts on the Hagberg Falling Number test. With the green revolution and the introduction of the *Rht* (*Reduced height*) dwarfing genes, LMA has evolved to a more stochastic expression^53^. In semi-dwarf germplasms LMA expression can vary between locations, between plants, and even between heads or spikelets from the same plant. It has been generally acknowledged that a cold shock between 20–25 days after anthesis can trigger LMA^54^. These conditions are necessary, but sometimes not sufficient, to trigger LMA thus making LMA detection and prediction very problematic. Historically, LMA is triggered by cold shock, but the nature of the stimulus and the mechanism underpinning this condition are ill-defined. Another group described in wheat the occurrence of high pI α-amylase on small set of United Kingdom genotypes following a heat shock^55,56^. The authors described a great variability in LMA occurrence triggered by either cold or heat shock. They associated this variability to the window of sensitivity described previously. However, a recent paper demonstrated than a prolonged and continuous cool maximum temperature regimen (23 °C/15 °C day/night) during grain development resulted in a more consistent LMA expression^29^. In 2014 and in United States Pacific West Coast, LMA was responsible for over 150 million $US loss to the growers in a single season. However, no cold shock was reported during this period^57^. In rice, accumulation of α-amylase has been described during grain developmental heat stress or more generally abiotic stress^58^. As a result, there is an emerging hypothesis that severe and rapid temperature changes may trigger LMA.

Although LMA is considered relatively recent grain quality defect worldwide^51^, our results suggest that the mechanism underpinning the LMA phenotype has been embedded into the genome of landraces for a long time. Using a landrace collection, we were able to demonstrate that heat stress can trigger LMA within a very wide range of both winter and spring varieties with highly variable climates of origin including Australia, Kazakhstan, Tunisia, Bosnia or Ethiopia.

With climate change leading to more unpredictable weather patterns, variation in rainfall and increased frequency of hot spells^19^ it is expected that PHS and LMA may occur more frequently. Our heat stress treatment, which mimics severe hot spells, appears to be a good assay to quickly identify both LMA and PHS resistant genotypes, and to evaluate the impact of heat shocks on grain size. Our results suggest that the traits we have examined are regulated independently and we have found genotypes that are insensitive to heat in relation to dormancy loss, or to LMA expression or to grain shrinkage (Table 2). However, it could be possible that germplasm displaying resistance to heat for three traits simultaneously exists when studying larger populations. In any case, given that genetic variability is observed in wheat, several approaches can be pursued in order to identify the genetic elements associated with the resistance to hot spells. The next target will be the utilization of genome wide association studies using populations such as the Vavilov collection to identify genetic markers linked to heat-resistance, which could then be used in breeding and tested in field conditions. Ultimately, it will allow the identification of the underlaying genes involved in heat tolerance for allele mining activities or gene editing approaches.

## Methods

### Plant material

The landraces and the commercial wheat (*Triticum aestivum*) cultivars used in this work were obtained from the Australian Winter Cereals Collection, Horsham, Victoria (Australia). Landraces were selected on the bases of their different geographical distribution and diverse morphology. Only spring or facultative genotypes were chosen to avoid vernalization treatments. The individual lines underwent two rounds of single seed descent in order to remove heterozygosity. Description of their accession numbers, origins and habits can be found in Table 1. Vavilov lines were kindly provided by Dr Lee Hickey (University of Queensland, Brisbane, Queensland, Australia) and their details can be found in Riaz et al.^30^.

### Growth conditions and heat shock treatment

Plants were grown in two Conviron growth chambers (model PGC20) in long day conditions (16 h light, 500 μmol m^−2^ s^−1^, Phillips TLD36 W/865 fluorescent tubes) and with a sine temperature regime reaching 20 °C at midday and 12 °C at midnight as in Barrero et al.^28^. This regime was chosen because it optimises the development of grain dormancy and because it is within the optimum temperature for grain filling in wheat^10^. In each cabinet genotypes were sown into four pots (20 cm diameter, one plant per pot) in a randomised design replicated in both cabinets. The plants in one of the cabinets were used as the control plants. In the second cabinet, all plants were tagged at anthesis and at 20 days post anthesis they were transferred into a third cabinet with a 36 °C at midday and 24 °C at midnight sine temperature regime for 10 days, and then returned to the original cabinet. At physiological maturity (using the collapse of the first node as a visual marker) two or three spikes per plant were harvested and dried at 37 ºC for 24 h and stored at – 20 ºC. The 10-day duration of the heat stress treatment was based on the 5-day treatment performed in Barrero et al.^28^ and we double it in this work in order to allow for differences in development and in sensitivity to heat in our diverse landraces.

### Germination assays

For dormancy studies, spikes harvested at maturity were hand-threshed and 20 grains per replicate were placed on 90 mm Petri dishes containing one 90 mm Whatman 598 filter paper and 5 mL of water. The plates were sealed with Parafilm and incubated at 20 ºC wrapped in two layers of aluminium foil. Germination was scored at 7 days, counting grains with emerged coleorhiza as germinated, and the percentage of germination was calculated. The check the growth conditions and heat treatment reproducibility, three landraces showing contrasting dormancy responses in the first experiment were chosen and the experiment was replicated with very similar results (Supplementary Fig. 2).

### LMA

LMA testing was performed on protein extracted from 10 mg wholemeal flour according to the method described by Verity et al.^59^ and Newberry et al.^60^. All spectrophotometric measurements were performed using a Thermo Scientific Multiskan Spectrum plate reader. LMA testing was restricted to 24 out of 28 landraces due to grain availability.

### Grain size analysis

Grain dimensions were measured in a flat scan using the GrainScan software^61^. Between 50 and 150 grains were analysed per sample. Due to yield limitation and grain availability for some landraces, grain size analyse were restricted to 26 out of 28 landraces.

### Diversity analysis

The panel of 28 wheat landraces was selected to represent global diversity in hexaploidy wheat. We assessed diversity of the selected landraces by comparison with a subset of 273 spring or semi-winter varieties from an established global wheat diversity set, the Vavilov wheat diversity panel^30^. Single nucleotide polymorphism (SNP) data were captured using the Illumina iSelect 90K SNP array^31^, at AgriBio, La Trobe University (Victoria). SNP data were filtered on minor allele frequency > 3.5% and missing data < 30%, resulting in 26,519 polymorphic SNPs for downstream analysis. This dataset has been deposited at the CSIRO Data Access Portal were can be publicly accessed (https://doi.org/10.25919/5f2365e6671d8). Comparison among varieties was performed based on SNP genotype data called from the 90K SNP array. Genomic marker data consisting of > 26K biallelic SNPs were applied using the dist() function in the base R package (v 3.5.1)^62^ to estimate pairwise Euclidean distance between varieties. To represent these distances in a visually informative way we subsequently applied a classical multiple dimensional scaling (MDS) algorithm to translate pairwise distances among varieties into a lower dimensionality space via principal components analysis (PCA), implementing the cmdscale() function in the base R package, with two dimensions. The first two orthogonal axes (coordinates) were then visualised using the ggplot2 R package^63^.

### Temperature modelling

Australia devotes about 18 million hectares to grow winter cereals (from Australian Bureau of Agricultural and Resource Economics and Sciences), although the number of hectares can vary year to year. Here, we investigated the long term mean of maximum temperatures for the months when the grain filling of winter cereals occurs in Australia (September to October) for three different periods. We obtained data from the Australian Bureau of Meteorology for two periods: baseline (1951–1980), contemporary data including the Australian Millennium drought (2003–2013) and finally we developed a predictive model for the future scenario (2021–2050). The meteorological data was obtained in raster format. The map was created in R^62^ using the Raster Package^64^ and we applied a mask where only pixels classified as ‘winter cereals’ (grey pixels in map in Fig. 1A) by the Australian Land Use and Management (ALUM) Classification system version 8 (https://data.gov.au/dataset/ds-dga-a7d50fb8-b353-4bb4-a7ca-1a2f38f44abc/details). The projected maximum temperatures for the period 2021–2050 were obtained from the Australian Earth System Model, Access 1.3^26^, based on the high carbon emissions Representative Concentration Pathway scenario (RCP 8.5) This scenario is equivalent to an increase in temperatures above 2 °C by 2050, which is the path we are currently tracking^65^.

## Supplementary information

Supplementary Legends.

Supplementary Figure 1.

Supplementary Figure 2.

## Data Availability

The datasets generated and analysed during the current study are included in this published article or have been deposited in a publicly accessible repository.
