# Supplementary material for: Evaluation of the impact of heat on wheat dormancy, late maturity α-amylase and grain size under controlled conditions in diverse germplasm
Source: Sci Rep. 2020 Oct 20;10:17800. doi: 10.1038/s41598-020-73707-8 (PMC7576155; doi:10.1038/s41598-020-73707-8)
Supplement: Supplementary file 1 — Supplementary Legends. [file 41598_2020_73707_MOESM1_ESM.docx]

**Evaluation of the impact of heat on wheat dormancy, late maturity alpha-amylase and grain size under controlled conditions in diverse germplasm**

Jose M. Barrero, Luciana Porfirio, Trijntje Hughes, Jing Chen, Shannon Dillon, Frank Gubler and Jean-Philippe F Ral

**SUPPLEMENTARY MATERIALS**

**Supplementary Figure 1. Effect of the heat treatment on the grain width and length.** Orange circles represent heat stressed samples. Mean values with their SEs are shown. nd: not determined.

**Supplementary Figure 2.** **Technical validation of the dormancy results for three selected land races.** We selected one genotype with heat-resistant dormancy (Aus7116), one line with heat-sensitive dormancy (Aus38554) and one with no dormancy at all (Aus9532). Results from this test show similar responses and rankings that in our main experiment.
